# Conversational Artificial Intelligence as a Source of Oral Health Information: A Cross-Sectional Study in a Romanian Population

**DOI:** 10.3390/dj14070424

**Published:** 2026-07-10

**Authors:** Marina Antoneta Pop, Abel Emanuel Moca, Mihai Porumb, Anca Porumb

**Affiliations:** 1 Doctoral School of Biomedical Sciences, University of Oradea, 1 Universitatii Street, 410087 Oradea, Romania; burynell23@yahoo.com; 2 Department of Dentistry, Faculty of Medicine and Pharmacy, University of Oradea, 4 Universității Street, 410087 Oradea, Romania; mihailive4321@gmail.com (M.P.); anca.porumb@yahoo.com (A.P.)

**Keywords:** artificial intelligence, oral health, large language models, dental behavior

## Abstract

**Background/Objectives:** Large language models have created new pathways for patients to access health information, yet little is known about how the general population uses these conversational artificial intelligence (AI) tools for oral health concerns. This study investigated patterns of AI use as a source of oral health information, the nature of data shared with these systems, users’ perceptions, and the impact on dental care-seeking behavior. **Methods:** A cross-sectional study was conducted among adults from Bihor County, Romania, using a structured 16-item online questionnaire distributed via social media, with eligibility restricted to individuals who had previously used conversational AI for oral health information. The final sample comprised 393 valid responses from this self-selected group of users. Fisher’s exact test and Z-tests with Bonferroni correction were applied (α = 0.05). **Results:** Most participants were female (68.2%), university-educated (52.9%), and lived in an urban setting (88.3%). Significant differences in patterns of AI use for oral health information were identified according to age, sex, and living environment (*p* < 0.001). Younger participants used AI more frequently, while older individuals perceived the information as less clear. The vast majority used AI for informational or preliminary guidance purposes, with very few treating it as a substitute for professional opinion. A relevant subset shared visual data (intraoral photographs or radiographs) with AI systems, raising data privacy concerns. Rural participants more frequently delayed dental visits and less often discussed AI-derived information with their dentist compared to urban participants. **Conclusions:** When used by the public as a source of oral health information, AI is increasingly adopted, with adoption shaped by age, sex, and socioeconomic context. These findings concern only this informational use and do not extend to other applications of AI in dentistry, such as diagnostic support, image analysis, or clinical decision-making. Dental professionals should proactively engage patients about their use of AI for oral health information to ensure that digitally obtained content is appropriately contextualized.

## 1. Introduction

In the past decade, AI has experienced rapid development, becoming one of the most transformative technologies of contemporary society [1]. Advances in machine learning and natural language processing have enabled the development of systems capable of analyzing large volumes of data and generating complex, contextually relevant responses in a manner increasingly similar to human communication [2]. Among these, large language models (LLMs) have attracted considerable interest from both the academic community and the general public [3]. Platforms such as ChatGPT (OpenAI, San Francisco, CA, USA), Gemini (Google LLC, Mountain View, CA, USA), and Copilot (Microsoft Corporation, Redmond, WA, USA) allow users to quickly obtain information and explanations through natural conversational interactions, facilitating access to knowledge across a wide range of fields, including healthcare and medicine [3,4,5].

The accessibility of these technologies is further enhanced by the widespread use of smartphones and the near-universal availability of internet connectivity [6]. In this context, patients increasingly turn to online resources to obtain health-related information, whether before visiting a physician or following a medical consultation. Traditionally, such information-seeking behavior has been carried out primarily through search engines such as Google (Google LLC, Mountain View, CA, USA) [7]. However, the emergence of conversational AI systems has introduced a new paradigm for accessing information, one in which users can formulate detailed questions, describe symptoms, or request personalized explanations [3,8]. As a result, AI is becoming, for many patients, a readily accessible source of medical information that requires no specialized equipment or technical expertise, but simply a standard mobile device [8,9].

The use of AI in seeking medical information carries both potential benefits and notable limitations [3]. On one hand, these tools offer rapid access to information, the ability to clarify medical concepts, and support in understanding symptoms or treatment options [3,10]. On the other hand, concerns have been raised regarding the accuracy of the information provided, the risk of symptom misinterpretation, and the possibility that patients may delay seeking professional care based on incomplete or incorrect responses [3,11]. In dentistry, these issues may be particularly relevant, as many oral conditions are initially assessed based on patient-reported symptoms or visual materials such as intraoral photographs and dental radiographs [12].

In recent years, several studies have evaluated the performance of AI models in generating medical or dental information and in supporting educational or clinical processes [3,12]. These studies have largely pursued two directions: assessing the technical accuracy, readability, or completeness of AI-generated responses against expert benchmarks, and evaluating the potential of AI to support clinicians in diagnostic, educational, or decision-support tasks. Recent reviews have further mapped the innovations, ethical considerations, and integration barriers associated with AI in dentistry, highlighting that its adoption raises not only technical but also ethical, legal, and acceptance-related challenges [13]. However, the existing literature has focused primarily on assessing these systems from the perspective of healthcare professionals, or on evaluating the accuracy of AI-generated responses. Comparatively little attention has been paid to how patients or the general population actually use these tools in real-world, unsupervised settings to seek information about oral health problems, what information they disclose to these systems, and how such information may influence their behavior in relation to dental care services [3,14]. Understanding this patient-facing dimension is clinically important. Unlike professional applications, which operate under expert oversight, patients interact with conversational AI without supervision, and the information they obtain may shape whether, when, and how they seek dental care. Characterizing these behaviors is therefore a necessary first step toward identifying potential risks and guiding how dental professionals should respond, an aspect that remains largely unexplored, particularly in dentistry.

Against this background, a clearer understanding is needed of how patients incorporate conversational AI into everyday health-seeking behavior, what types of oral health concerns prompt its use, what information is entered into these systems, and what perceptions and levels of trust users associate with AI-generated content. It should be emphasized that AI in dentistry encompasses a broad spectrum of distinct applications, including diagnostic support, radiographic and image analysis, clinical decision support, risk prediction, patient triage, pharmacological support, education, administrative automation, personalized prevention, and professional training [5]. The present study does not address these clinical or professional applications, but rather focuses exclusively on the use of conversational AI tools by the general public as a source of oral health information. To date, this behavior has been examined almost exclusively in other national and healthcare contexts, and data on how the Romanian population uses conversational AI for oral health information remain scarce. Bihor County, which includes both a major urban center and extensive surrounding rural areas, provides a suitable setting in which to explore this question across different living environments. Therefore, the aim of the present study was to investigate how individuals use conversational AI tools to obtain information about dental conditions, the nature of the information they provide to these systems, their perceptions regarding usefulness and reliability, and the potential impact on their dental care-seeking behavior.

## 2. Materials and Methods

### 2.1. Study Design and Participants

This observational, cross-sectional study was conducted among the general adult population of Bihor County, Romania. Data were collected between 1 February and 1 April 2026 using a structured questionnaire administered online via Google Forms (Google LLC, Mountain View, CA, USA). The questionnaire link was distributed through social media platforms to reach a broad segment of the population.

Participation was voluntary and anonymous, with each respondent completing a single questionnaire. Inclusion criteria comprised an age of ≥18 years, residence in Bihor County, Romania, and prior use of at least one conversational AI tool to obtain oral health-related information. Incomplete questionnaires were excluded from the analysis. Because participation required prior use of a conversational AI tool for oral health information and recruitment was conducted through social media, the resulting sample was a self-selected group of existing conversational AI users. Consequently, the study was designed to characterize behaviors and perceptions within this population of users, and not to estimate the prevalence of conversational AI use in the general adult population. The study was reported in accordance with the Strengthening the Reporting of Observational Studies in Epidemiology (STROBE) guidelines.

### 2.2. Questionnaire and Variables

A structured questionnaire, developed in Romanian, was used to assess how individuals use conversational AI tools to obtain oral health-related information. The instrument consisted of 16 items organized into five thematic sections, each comprising predefined response options appropriate to the type of information assessed. Most items were formulated as single-choice questions, while perceptions and attitudes were measured using 5-point Likert scales. The sections were as follows:

- Section I—Sociodemographic characteristics: age group, sex, educational level, living environment, and frequency of dental visits;
- Section II—Context of AI use: frequency and timing of AI use in relation to dental consultations, type of dental problem, and main purpose of use;
- Section III—Type of information provided to AI: nature of data entered (symptoms, images, personal data), use of image-based interpretation, and the role attributed to AI (informational, preliminary guidance, or substitute for professional opinion);
- Section IV—Attitudes and trust: perceived accuracy, clarity, and usefulness of the information provided, as well as its potential to induce anxiety;
- Section V—Impact on behavior and the dentist–patient relationship: influence on dental care-seeking behavior, communication with the dentist, and overall perceived utility.

The questionnaire was developed by the research team based on a review of the existing literature on the use of conversational AI for health information and on the specific objectives of the study. To assess content and face validity, the draft instrument was reviewed by two general dental practitioners, who evaluated the relevance, comprehensiveness, and appropriateness of the items in relation to the study aims; their qualitative comments were used to refine item content and wording. The questionnaire was then pretested on a pilot group of 15 respondents drawn from the target population, who completed the instrument and subsequently provided verbal feedback through informal discussion regarding the clarity, comprehensibility, and interpretation of the items. Based on this feedback, ambiguous items were rephrased and the response options of several questions were revised to better reflect the range of possible answers. Because the pilot phase was intended to improve clarity and face validity rather than to establish psychometric properties, no formal reliability or construct validity testing was performed, and the responses collected during the pilot phase were not included in the final analysis.

Before completing the questionnaire, participants were provided with introductory information regarding the study’s purpose, the anonymous and voluntary nature of participation, the estimated completion time (approximately 4–5 min), and the exclusive use of data in aggregated form for scientific purposes. Respondents were informed that there were no correct or incorrect answers and that they could withdraw at any time without consequences. Completion of the questionnaire implied informed consent for the use of data for research purposes, as well as confirmation of prior conversational AI use for oral health-related information.

Each questionnaire item was analyzed individually rather than incorporated into a composite score, in order to preserve the specificity of the distinct dimensions investigated (namely patterns of AI use, user attitudes, and behavioral impact). Given the exploratory nature of the study and the absence of a validated composite index for the variables assessed, no overall score was constructed.

### 2.3. Sample Size

The minimum required sample size was estimated a priori using Python (version 3.14.5, Python Software Foundation, Wilmington, DE, USA), assuming a 95% confidence level and a margin of error of 5%. In the absence of prior estimates for the proportion of the variables of interest, a conservative proportion of *p* = 0.5 was applied, as this maximizes variability and yields the largest required sample size. Based on these parameters, the minimum estimated sample size was approximately 384 participants. After applying the inclusion and exclusion criteria, the final sample consisted of 393 valid questionnaires.

### 2.4. Statistical Analysis

All statistical analyses were performed using IBM SPSS Statistics, version 25 (IBM Corp., Armonk, NY, USA). Data organization and presentation were carried out using Microsoft Office Excel and Word 2024 (Microsoft Corp., Redmond, WA, USA). Qualitative variables were expressed as absolute frequencies and percentages. Differences between groups were assessed using Fisher’s exact test, and Z-tests with Bonferroni correction were subsequently applied to further explore significant associations identified in contingency tables. The level of statistical significance was set at α = 0.05.

### 2.5. Ethical Considerations

The study was conducted in accordance with the ethical principles of the Declaration of Helsinki (1964) and its subsequent revisions. The research protocol was approved by the Ethics Committee of the University of Oradea (Approval No. 70/30.01.2026). Participation was voluntary and anonymous, and no financial or other incentives were offered. Informed consent was considered implied through the voluntary completion of the questionnaire, following the provision of information regarding the study’s purpose and the exclusive scientific use of the collected data.

## 3. Results

All analyses refer to a self-selected sample of existing conversational AI users and describe patterns within this group rather than its frequency in the general population. The demographic characteristics of the analyzed participants are presented in Table 1. Most participants were aged 35–44 years (36.6%, *n* = 144), female (68.2%, *n* = 268), held a university degree (52.9%, *n* = 208), and lived in urban areas (88.3%, *n* = 347). Regarding dental attendance, the majority reported visiting the dental office regularly (at least once per year) (63.9%, *n* = 251).

As shown in Table 2, multiple significant age-related differences were identified across all analyzed variables (*p* < 0.001). Younger participants (18–24 years) reported more frequent AI use (56.1%, *n* = 37), while those aged ≥55 years were more likely to use AI only once (63.6%, *n* = 14). AI use before and after dental visits was more common among younger groups (65.2%, *n* = 43 in 18–24), whereas use exclusively after visits was more frequent in older participants (27.3%, *n* = 6 in ≥55).

Regarding reasons for AI consultation, dental pain was predominantly reported by individuals aged 35–44 (67.4%, *n* = 97), while gum-related concerns were more frequent among younger participants (59.1%, *n* = 39 in 18–24). Diagnostic purposes were more common in the youngest and middle-aged groups (62.1%, *n* = 41 in 18–24; 60.7%, *n* = 54 in 45–54), whereas home management advice was more frequently sought by older individuals (50%, *n* = 11 in ≥55).

In terms of interaction with AI, most younger participants provided only symptom descriptions (92.4%, *n* = 61 in 18–24), while sending intraoral images or radiographs was more frequent in older groups, particularly those aged 45–54 (44.4%, *n* = 40). Similarly, the use of AI for medical imaging interpretation was higher in this age group (44.1%, *n* = 40), while younger participants largely did not use AI for this purpose (93.9%, *n* = 62 in 18–24).

Perceptions of AI also varied significantly. Younger participants more often considered the information clear and useful (53%, *n* = 35 in 18–24), whereas older individuals reported unclear information more frequently (45.5%, *n* = 10 in ≥55). High or very high agreement regarding AI usefulness was observed especially in younger and middle-aged groups (72.7%, *n* = 48 high agreement in 18–24; 56.3%, *n* = 81 very high in 35–44).

Finally, discussions with dentists about AI-derived information were more frequent among participants aged 35–44 (68.1%, *n* = 98), and overall positive perceptions of AI usefulness were highest in younger and middle-aged groups (56.1%, *n* = 37 in 18–24; 59%, *n* = 85 in 35–44).

As shown in Table 3, significant gender-related differences were identified for several analyzed variables (*p* < 0.001). Men reported using AI occasionally more frequently than women (42.4%, *n* = 53 vs. 28.7%, *n* = 77), whereas very frequent use was more common among women (15.7%, *n* = 42 vs. 0.8%, *n* = 1).

Regarding reasons for AI consultation, men more frequently used AI for dental pain (64.8%, *n* = 81 vs. 24.3%, *n* = 65) and for assessing severity (32%, *n* = 40 vs. 10.1%, *n* = 27), while women more often sought information on cavities/dental sensitivity (26.9%, *n* = 72 vs. 10.4%, *n* = 13), periodontal issues (29.1%, *n* = 78 vs. 8.0%, *n* = 10) and home management strategies (25.7%, *n* = 69 vs. 13.6%, *n* = 17).

In terms of interaction with AI, men predominantly provided only symptom descriptions (92.8%, *n* = 116 vs. 67.9%, *n* = 182), whereas women were more likely to share additional data, including intraoral photographs (17.2%, *n* = 46 vs. 2.4%, *n* = 3), or combined information (12.3%, *n* = 33 vs. 2.4%, *n* = 3). Similarly, women more frequently used AI for X-ray interpretation (16.4%, *n* = 44 vs. 4.0%, *n* = 5), while men more often submitted combined imaging data (25.6%, *n* = 32 vs. 9.3%, *n* = 25).

Differences were also observed in perceived usefulness. Women more frequently considered AI information as mostly correct (62.7%, *n* = 168 vs. 34.4%, *n* = 43) and showed higher levels of agreement regarding usefulness (54.9%, *n* = 147 vs. 22.4%, *n* = 28) and ease of understanding (64.2%, *n* = 172 vs. 30.4%, *n* = 38). In contrast, men more often rated AI outputs as very clear and useful (51.2%, *n* = 64 vs. 19.8%, *n* = 53) and expressed very high agreement regarding usefulness (48.8%, *n* = 61 vs. 10.4%, *n* = 28), ease of understanding (51.2%, *n* = 64 vs. 13.1%, *n* = 35), and the ability of AI to identify emergencies (48%, *n* = 60 vs. 10.3%, *n* = 28).

Finally, women more frequently reported neutral attitudes regarding the potential of AI to induce anxiety (64.2%, *n* = 172 vs. 38.4%, *n* = 48), while men more often expressed very high concern in this regard (27.2%, *n* = 34 vs. 4.1%, *n* = 11).

As shown in Table 4, significant differences were observed according to living environment (*p* < 0.05). It should be noted that the rural subgroup was small (*n* = 46) relative to the urban subgroup (*n* = 347), and the rural percentages below are therefore based on small absolute numbers. Rural participants more frequently reported single-time AI use (50%, *n* = 23 vs. 27.1%, *n* = 94) and use just before or after the dental visit (60.9%, *n* = 28 and 19.6%, *n* = 9), whereas urban participants more often used AI both before and after visits (57.3%, *n* = 199) and reported more frequent use (28.8%, *n* = 100 vs. 6.5%, *n* = 3).

Urban participants more commonly used AI for diagnostic purposes (50%, *n* = 174 vs. 26.1%, *n* = 12) and shared radiographic data (14%, *n* = 47) vs. 0%, *n* = 0), while rural participants relied more on symptom descriptions, intraoral images, and home management advice (41.3%, *n* = 19). Rural participants were also more likely to delay dental visits (13%, *n* = 6 vs. 2.3%, *n* = 8), whereas urban participants more often reported no change (85.6%, *n* = 297) and more frequently discussed AI information with their dentist (59.7%, *n* = 207) vs. 10.9%, *n* = 5).

Overall, rural participants tended to consider AI useful (39.1%, *n* = 18), while urban participants more often rated it as very useful (49.9%, *n* = 173).

To provide an integrated overview and to reduce the risk of fragmented interpretation arising from the large number of individual bivariate comparisons, the most clinically relevant significant associations identified across age, gender, and living environment are summarized in Table 5. These associations were selected on the basis of their clinical and patient-safety relevance, including the sharing of visual data with AI, the use of AI as a substitute for professional opinion, the perceived potential of AI to induce anxiety, delayed dental attendance, and discussion of AI-derived information with the dentist.

## 4. Discussion

The study investigated how the adult population of Bihor County, Romania, uses conversational AI tools to obtain oral health-related information, while also examining the types of information provided to these systems, users’ perceptions, and the potential impact on dental care-seeking behavior. Because participation was restricted to individuals who had already used conversational AI for this purpose and recruitment was conducted through social media, the findings describe behaviors and perceptions within this group of users and do not estimate the prevalence of conversational AI use in the general adult population. The findings reveal a heterogeneous usage profile, with significant differences according to participants’ age, sex, and living environment. It should be emphasized that the present study captured participants’ self-reported perceptions and behaviors. It did not assess the accuracy of the information obtained from conversational AI, its effect on clinical outcomes, or whether its use directly caused any change in dental care-seeking behavior. The interpretations that follow should therefore be read as associations and perceptions reported by users rather than as demonstrated clinical effects.

The demographic profile of the study sample reflects a pattern of digital technology adoption consistent with that reported in the existing literature. The overrepresentation of university-educated individuals and urban residents suggests that the use of conversational AI for oral health information is not uniform across the general population, but rather follows the well-established gradients of the digital divide. The distribution is explained by documented differences in internet access and digital competencies between urban and rural areas in Romania, as well as by the well-established association between educational level and the capacity to effectively use digital health tools [15,16]. It should be emphasized, however, that the sample was predominantly female, urban, and university-educated, and that rural participants were few (*n* = 46, 11.7%). This imbalance limits the representativeness of the sample and means that subgroup comparisons, particularly those involving the rural group, are based on small numbers and should be interpreted with caution.

The inverse relationship between age and frequency of AI use observed in our study is consistent with trends reported in the existing literature, which confirm that the adoption of medical chatbots follows a well-documented generational gradient [16]. This distribution does not necessarily reflect a lack of interest among older age groups, but rather differences in familiarity with conversational interfaces and in the level of trust placed in these technologies [17]. In this context, our data suggest that, although conversational AI is becoming an increasingly accessible source of oral health information for those who use it, its benefits appear to be unevenly leveraged, with younger individuals remaining the primary active users of these tools.

An encouraging finding from a patient safety perspective is that the self-reported use of AI as a substitute for professional medical opinion was extremely rare in the study sample, with the vast majority of participants attributing an informational or preliminary guidance role to these tools. This tendency suggests that users generally maintain a clear distinction between digital information-seeking and the medical act itself, perceiving AI as a complementary instrument to the dental consultation rather than a replacement for it. These results are consistent with the existing literature, which indicates that even among active users of medical chatbots, the majority continue to seek professional validation of the information obtained [18].

Although verbal description of symptoms represented the predominant mode of interaction with AI systems, a relevant subset of participants also provided visual data (such as intraoral photographs or dental radiographs), a behavior more frequently observed among middle-aged groups and female participants. This practice raises legitimate concerns from a data protection perspective, as general-purpose conversational AI platforms are not designed to handle sensitive medical information and do not offer explicit guarantees regarding the confidentiality or security of transmitted data [19,20]. Unlike certified medical systems, these tools are not subject to healthcare-specific regulations, meaning that users who share medical images may be exposed to privacy risks in the event of a data breach, often without being aware of this [20].

A further and particularly high-risk dimension of patient-facing AI use concerns pharmacological queries. Beyond interpreting symptoms or images, patients may turn to conversational AI to obtain advice on prescribed drugs, chronic therapies, and potential drug–drug interactions. This is especially relevant in dentistry and oral surgery, where commonly prescribed perioperative agents, such as analgesics, antibiotics, corticosteroids, and non-steroidal anti-inflammatory drugs, may interact with a patient’s chronic medications and lead to clinically significant adverse events. Recent evidence indicates that current large language models still exhibit clinically relevant limitations in detecting drug–drug interactions in dental therapy; although they can achieve high sensitivity, they tend to generate a substantial proportion of false-positive alerts and to misjudge interaction severity, and should therefore be regarded as supervised decision-support tools rather than substitutes for professional judgment [21]. More broadly, the need for appropriate guardrails when large language models are used in pharmacovigilance and other safety-critical medical settings has been emphasized, given their propensity to produce fabricated or inaccurate information that could result in patient harm [22]. These concerns are directly relevant to the present findings, as a proportion of participants reported using conversational AI before dental visits and indicated that AI-derived information could influence their care-seeking behavior. In this context, there is a tangible risk that a patient might ask an AI tool whether a prescribed analgesic, antibiotic, corticosteroid, or NSAID is safe in combination with their chronic medication and, on the basis of the response, decide to delay, interrupt, or modify a therapy without consulting a clinician. AI-derived pharmacological advice cannot currently be considered sufficiently reliable to guide such decisions without professional validation, and patients should be explicitly cautioned against making unsupervised medication decisions based on conversational AI, with dental or medical verification remaining essential.

Perceptions regarding the usefulness and accuracy of AI-generated information varied significantly according to age, a pattern more appropriately interpreted through the lens of differential digital competencies rather than differences in the actual quality of the generated responses, which the present study did not assess. The ability to formulate precise questions and to navigate conversational interfaces effectively may directly influence the quality of interaction with conversational AI systems and, consequently, the perceived relevance of the information obtained. In this regard, the advantage of younger generations in positively evaluating AI is consistent with the existing literature, which documents a deeper digital integration among this demographic, shaped by early and continuous exposure to digital platforms and algorithms [23,24].

A clinically noteworthy finding is participants’ self-reported perception that conversational AI may induce unwarranted anxiety regarding oral health problems, a perception more pronounced among male participants. This observation falls within the broader framework of cyberchondria, defined as the tendency to develop exaggerated health concerns following online searches for medical information, which is being increasingly documented in the context of unguided access to digital medical content [25]. Although conversational AI can facilitate rapid access to information, the absence of a professional interpretive framework may transform an information-seeking behavior into a source of distress, with potential consequences on patients’ dental care-seeking behavior [26].

From a clinical perspective, the data from our study suggest that conversational AI use does not substantially alter the self-reported dental care-seeking behavior of the majority of patients; however, its perceived impact is not uniform across the sample. Of particular concern is the self-reported tendency to delay dental consultations, more frequently reported among rural participants, where geographic and economic barriers to dental care access are well documented [27,28]. This observation should, however, be interpreted with caution, as it is based on a small rural subgroup, in which delayed visits were reported by only six participants (13%). Moreover, the cross-sectional, self-reported design does not permit causal inference: it cannot be determined whether conversational AI use contributed to these delays or whether both simply reflect the same underlying barriers to dental care access. The finding is therefore exploratory and hypothesis-generating rather than conclusive. In these communities, conversational AI may be used more as a substitute for professional consultation than as a complementary tool. The significant gap in the rate of discussions with dental practitioners regarding AI-derived information between urban and rural participants suggests that the potential benefits of these tools may be unevenly leveraged, depending on the user’s socioeconomic context. The existing literature has highlighted that inequality in access to qualified healthcare services between urban and rural settings remains one of the main challenges facing healthcare systems in developing countries [29,30], and the findings of the present study may suggest that this disparity extends to the way AI-based sources of oral health information are utilized.

A positive finding relevant to the patient–practitioner relationship is that a substantial proportion of participants used AI as a tool for consultation preparation, which was reflected both in their ability to formulate more precise questions and in their willingness to discuss AI-derived information with their dental practitioner. This tendency is consistent with data from the international literature, which documents similar behavior among medical chatbot users [31,32]. However, the fact that nearly half of the participants did not discuss AI-obtained information with their dentist raises an important clinical concern, namely that of decisions being made on the basis of professionally unverified information. This reality underscores the need for dental practitioners to adopt a proactive approach regarding their patients’ use of conversational AI, integrating this discussion into the consultation and providing an appropriate framework for the interpretation and, where necessary, the correction of information obtained from digital sources [33].

The present study has several limitations that should be considered when interpreting the findings. First, the observational and cross-sectional design does not allow for the establishment of causal relationships. The sample was recruited through social media platforms, which may introduce a selection bias in favor of individuals with internet access and a higher level of familiarity with digital technology. Furthermore, the study was limited to the population of Bihor County, Romania, which may restrict the external validity of the findings. In addition, the study addressed only the use of conversational AI as a source of oral health information for the public and did not evaluate other applications of AI in dentistry, such as diagnostic support, image analysis, clinical decision-making, or risk prediction; accordingly, the findings should not be generalized beyond this specific informational use. Moreover, questionnaire responses reflect participants’ perceptions and self-assessments, which may be influenced by subjective factors or social desirability bias. Finally, the study relied on a purpose-designed questionnaire that underwent content and face validity assessment and pilot testing but was not formally validated; its psychometric properties, including reliability and construct validity, were not established, which should be taken into account when interpreting the findings.

## 5. Conclusions

Conversational AI has become an increasingly relevant source of oral health information for the general population, with adoption patterns shaped by age, sex, and socioeconomic context. Because the study describes a self-selected sample of conversational AI users rather than the general population, these findings characterize how such users engage with these tools and do not indicate how widespread this use is among adults overall. While most users attribute a complementary rather than substitutive role to these tools, important concerns remain regarding data privacy, the risk of health-related anxiety, and possible inequalities in the distribution of benefits across urban and rural communities, although the latter observation is based on a small rural subgroup and requires confirmation in larger and more balanced samples. The potential of conversational AI to support consultation preparation and improve patient–practitioner communication represents a valuable opportunity that dental professionals should actively leverage. Proactive engagement with patients regarding their use of AI as a source of oral health information, combined with appropriate guidance on the critical evaluation of digitally obtained information, is essential to ensure that this informational use of AI contributes positively to oral health outcomes. These conclusions pertain solely to the use of conversational AI as a source of oral health information for the public and do not extend to other applications of AI in dentistry.

## Figures and Tables

**Table 1:** Demographic characteristics of the analyzed participants.

Parameter	Value	
	No.	Percentage
**Age (years)**		
18–24	66	16.8%
25–34	71	18.1%
35–44	144	36.6%
45–54	90	22.9%
≥55	22	5.6%
**Gender**		
Female	268	68.2%
Male	125	31.8%
**Education**		
Secondary	2	0.5%
High school	122	31.0%
Post-secondary	15	3.8%
University	208	52.9%
Postgraduate	46	11.7%
**Environment**		
Urban	347	88.3%
Rural	46	11.7%
**Dental visits**		
Only for pain/emergency	37	9.4%
Occasionally	105	26.7%
Regularly (≥1/year)	251	63.9%

**Table 2:** Distribution of the participants according to the answers in the survey and age.

Response (No., %)	Age (Years)					*p* *
	18–24	25–34	35–44	45–54	≥55	
Item 6—How often have you used AI to obtain information related to dental health?						
Once	10 (15.2%)	33 (46.5%)	36 (25.0%)	24 (26.7%)	14 (63.6%)	<0.001
A few times	19 (28.8%)	32 (45.1%)	52 (34.7%)	23 (25.6%)	6 (27.3%)	
Frequently	37 (56.1%)	4 (5.6%)	57 (39.6%)	4 (4.4%)	1 (4.5%)	
Very frequently	0 (0.0%)	2 (2.8%)	1 (0.7%)	39 (43.3%)	1 (4.5%)	
Item 7—When did you use AI?						
Just before visit	21 (31.8%)	45 (63.4%)	43 (29.9%)	31 (34.4%)	12 (54.5%)	<0.001
Only after visit	2 (3.0%)	7 (9.9%)	10 (6.9%)	8 (8.9%)	6 (27.3%)	
Before and after visit	43 (65.2%)	19 (26.8%)	91 (63.2%)	51 (56.7%)	4 (18.2%)	
Item 8—For which dental condition did you use AI?						
Dental pain	11 (16.7%)	18 (25.4%)	97 (67.4%)	15 (16.5%)	5 (22.7%)	<0.001
Cavity/sensitivity	9 (13.6%)	16 (22.5%)	17 (11.8%)	43 (47.3%)	0 (0.0%)	
Gums/periodontal issues	39 (59.1%)	12 (16.9%)	13 (9%)	15 (16.5%)	9 (40.9%)	
Oral lesions	1 (1.5%)	8 (11.3%)	1 (0.7%)	4 (4.4%)	2 (9.1%)	
Other	6 (9.1%)	17 (23.9%)	16 (11.1%)	13 (15.4%)	6 (27.3%)	
Item 9—What was the purpose of using AI?						
Diagnostic	41 (62.1%)	21 (29.6%)	64 (44.4%)	54 (60.7%)	5 (22.7%)	<0.001
Severity assessment	7 (10.6%)	14 (19.7%)	38 (26.4%)	5 (5.6%)	3 (13.6%)	
Emergency consultation	1 (1.5%)	1 (1.4%)	3 (2.1%)	1 (1.1%)	0 (0.0%)	
At home temporary solutions	10 (15.2%)	22 (31.0%)	25 (17.4%)	18 (20.2%)	11 (50.0%)	
Treatment options	7 (10.6%)	13 (18.3%)	14 (9.7%)	12 (12.4%)	3 (13.6%)	
Item 10—What type of information did you send to the AI?						
Only symptoms details	61 (92.4%)	59 (83.1%)	113(78.5%)	47 (52.2%)	18 (81.8%)	<0.001
Intraoral photos	3 (4.5%)	1 (1.4%)	4 (2.8%)	40 (44.4%)	1 (4.5%)	
Dental X-rays	1 (1.5%)	4 (5.6%)	1 (0.7%)	2 (2.2%)	0 (0.0%)	
Personal ID	1 (1.5%)	0 (0.0%)	0 (0.0%)	0 (0.0%)	1 (4.5%)	
All of the above	0 (0.0%)	7 (9.9%)	26 (18.1%)	1 (1.1%)	2 (9.1%)	
Item 11—Have you used AI for the interpretation of dental images?						
On X-rays	1 (1.5%)	5 (7.0%)	2 (1.4%)	40 (44.1%)	0 (0.0%)	<0.001
On intraoral photos	2 (3.0%)	5 (7.0%)	2 (1.4%)	0 (0.0%)	1 (4.3%)	
Both	1 (1.5%)	2 (2.8%)	51 (35.4%)	1 (1.1%)	2 (8.7%)	
Did not use images	62 (93.9%)	59 (83.1%)	89 (61.8%)	49 (54.8%)	20 (87.0%)	
Item 12—What was AI mainly used for?						
General information	56 (84.8%)	50 (70.4%)	72 (50.0%)	79 (87.8%)	14 (65.2%)	<0.001
Preliminary orientation	4 (6.1%)	7 (9.9%)	32 (22.2%)	5 (5.6%)	6 (26.1%)	
Suspicion confirmation	5 (7.6%)	13 (18.3%)	37 (25.7%)	2 (2.2%)	1 (4.3%)	
Substitution of medical opinion	1 (1.5%)	1 (1.4%)	3 (2.1%)	4 (4.4%)	1 (4.3%)	
Item 13—How would you evaluate the information provided by AI?						
Completely wrong	0 (0.0%)	1 (1.4%)	1 (0.7%)	0 (0.0%)	0 (0.0%)	<0.001
Partially wrong	2 (3.0%)	3 (4.2%)	1 (0.7%)	2 (2.2%)	1 (4.5%)	
Unclear	4 (6.1%)	14 (19.7%)	12 (8.3%)	14 (15.6%)	10 (45.5%)	
Mostly correct	25 (37.9%)	45 (63.4%)	64 (44.4%)	67 (74.4%)	10 (45.5%)	
Very clear and useful	35 (53.0%)	8 (11.3%)	66 (45.8%)	7 (7.8%)	1 (4.5%)	
Item 14—To what extent do you agree that AI provides useful information about dental conditions?						
Total disagreement	0 (0.0%)	1 (1.4%)	3 (2.1%)	0 (0.0%)	1 (4.5%)	<0.001
Disagree	1 (1.5%)	2 (2.8%)	0 (0.0%)	4 (4.4%)	1 (4.5%)	
Neutral	17 (25.8%)	33 (46.5%)	25 (17.4%)	29 (32.2%)	11 (50.0%)	
Agree	48 (72.7%)	31 (43.7%)	35 (24.3%)	54 (60.0%)	8 (36.5%)	
Total agreement	0 (0.0%)	4 (5.6%)	81 (56.3%)	3 (3.3%)	1 (4.5%)	
Item 15—To what extent do you agree that the information provided by AI is easy to understand?						
Total disagreement	0 (0.0%)	1 (1.4%)	3 (2.1%)	1 (1.1%)	0 (0.0%)	<0.001
Disagree	1 (1.5%)	2 (2.8%)	1 (0.7%)	0 (0.0%)	1 (4.5%)	
Neutral	8 (12.1%)	17 (23.9%)	15 (10.4%)	21 (23.3%)	12 (54.5%)	
Agree	54 (81.8%)	45 (63.4%)	40 (27.8%)	64 (71.1%)	8 (36.5%)	
Total agreement	3 (4.5%)	6 (8.5%)	85 (59%)	4 (4.4%)	1 (4.5%)	
Item 16—To what extent do you agree that AI helped you determine whether your dental problem was urgent?						
Total disagreement	1 (1.5%)	4 (5.6%)	6 (4.2%)	2 (2.2%)	2 (9.1%)	<0.001
Disagree	1 (1.5%)	9 (12.7%)	8 (5.6%)	5 (5.6%)	3 (13.6%)	
Neutral	12 (18.2%)	27 (38%)	18 (12.5%)	65 (72.2%)	12 (54.5%)	
Agree	52 (78.8%)	26 (36.6%)	30 (20.8%)	16 (17.8%)	5 (22.7%)	
Total agreement	0 (0.0%)	5 (7.0%)	82 (56.9%)	2 (2.2%)	0 (0.0%)	
Item 17—To what extent do you agree that the use of AI may induce unnecessary anxiety regarding dental problems?						
Total disagreement	1 (1.5%)	3 (4.2%)	8 (5.6%)	2 (2.2%)	3 (13.6%)	<0.001
Disagree	4 (6.1%)	10 (14.1%)	10 (6.9%)	10 (11.1%)	3 (13.6%)	
Neutral	47 (71.2%)	30 (42.3%)	74 (51.4%)	61 (67.8%)	8 (36.4%)	
Agree	11 (16.7%)	23 (32.4%)	20 (13.9%)	14 (15.6%)	6 (27.3%)	
Total agreement	3 (4.5%)	5 (7.0%)	32 (22.2%)	3 (3.3%)	2 (9.1%)	
Item 18—How did using AI influence the timing of your dental consultation?						
Scheduled sooner	7 (10.6%)	16 (22.5%)	16 (11.2%)	10 (11.1%)	2 (8.7%)	0.188
Delayed	1 (1.5%)	4 (5.6%)	5 (3.5%)	2 (2.2%)	2 (8.7%)	
Unchanged	58 (87.9%)	51 (71.8%)	122(85.3%)	78 (86.7%)	19 (82.6%)	
Item 19—To what extent do you agree that AI helped you formulate better questions for your dentist?						
Total disagreement	3 (4.5%)	3 (4.2%)	9 (6.3%)	2 (2.2%)	5 (22.7%)	<0.001
Disagree	1 (1.5%)	3 (4.2%)	3 (2.1%)	3 (3.3%)	0 (0.0%)	
Neutral	11 (16.7%)	28 (39.4%)	19 (13.2%)	23 (25.6%)	9 (40.9%)	
Agree	16 (24.2%)	33 (46.5%)	60 (41.7%)	59 (65.6%)	7 (31.8%)	
Total agreement	35 (53.0%)	4 (5.6%)	53 (36.8%)	3 (3.3%)	1 (4.5%)	
Item 20—Did you discuss the information obtained from AI with your dentist?						
No	25 (37.9%)	49 (69.0%)	46 (31.9%)	45 (50.0%)	15 (68.2%)	<0.001
Yes	41 (62.1%)	22 (31.0%)	98 (68.1%)	45 (50.0%)	7 (31.8%)	
Item 21—Overall, how would you rate the use of AI for dental problems?						
Confusing	1 (1.5%)	2 (2.8%)	3 (2.1%)	2 (2.2%)	3 (13.6%)	<0.001
Low usefulness	3 (4.5%)	10 (14.1%)	8 (5.6%)	11 (12.2%)	3 (13.6%)	
Neutral	12 (18.2%)	18 (25.4%)	17 (11.8%)	19 (21.1%)	9 (40.9%)	
Useful	13 (19.7%)	29 (40.8%)	31 (21.5%)	14 (15.6%)	5 (22.7%)	
Very useful	37 (56.1%)	12 (16.9%)	85 (59.0%)	44 (48.9%)	2 (9.1%)	

* Fisher’s Exact Test.

**Table 3:** Distribution of the participants according to the answers in the survey and gender.

Response (No., %)	Gender		*p* *
	Female	Male	
Item 6—How often have you used AI to obtain information related to dental health?			
Once	77 (28.7%)	40 (32.0%)	<0.001
A few times	77 (28.7%)	53 (42.4%)	
Frequently	72 (26.9%)	31 (24.8%)	
Very frequently	42 (15.7%)	1 (0.8%)	
Item 7—When did you use AI?			
Just before visit	105 (39.2%)	47 (37.6%)	0.915
Only after visit	23 (8.6%)	10 (8.0%)	
Before and after visit	140 (52.2%)	68 (54.4%)	
Item 8—For which dental condition did you use AI?			
Dental pain	65 (24.3%)	81 (64.8%)	<0.001
Cavity/sensitivity	72 (26.9%)	13 (10.4%)	
Gums/perirodontal issues	78 (29.1%)	10 (8.0%)	
Oral lesions	9 (3.4%)	7 (5.6%)	
Other	44 (16.4%)	14 (11.2%)	
Item 9—What was the purpose of using AI?			
Diagnostic	134 (50.0%)	52 (41.6%)	<0.001
Severity assessment	27 (10.1%)	40 (32.0%)	
Emergency consultation	5 (1.9%)	1 (0.8%)	
At home temporary solutions	69 (25.7%)	17 (13.6%)	
Treatment options	33 (12.2%)	15 (12.0%)	
Item 10—What type of information did you send to the AI?			
Only symptoms details	182 (67.9%)	116 (92.8%)	<0.001
Intraoral photos	46 (17.2%)	3 (2.4%)	
Dental X-rays	5 (1.9%)	3 (2.4%)	
Personal ID	2 (0.7%)	0 (0.0%)	
All of the above	33 (12.3%)	3 (2.4%)	
Item 11—Have you used AI for the interpretation of dental images?			
On X-rays	44 (16.4%)	5 (4.0%)	<0.001
On intraoral photos	8 (3.0%)	2 (1.6%)	
Both	25 (9.3%)	32 (25.6%)	
Did not used images	191 (71.3%)	86 (68.8%)	
Item 12—What was AI mainly used for?			
General information	195 (72.8%)	76 (60.8%)	<0.001
Preliminary orientation	46 (17.2%)	8 (6.4%)	
Suspicion confirmation	22 (8.2%)	36 (28.8%)	
Substitution of medical opinion	5 (1.8%)	5 (4.0%)	
Item 13—How would you evaluate the information provided by AI?			
Completely wrong	2 (0.7%)	0 (0.0%)	<0.001
Partially wrong	6 (2.2%)	3 (2.4%)	
Unclear	39 (14.6%)	15 (12.0%)	
Mostly correct	168 (62.7%)	43 (34.4%)	
Very clear and useful	53 (19.8%)	64 (51.2%)	
Item 14—To what extent do you agree that AI provides useful information about dental conditions?			
Total disagreement	4 (1.5%)	1 (0.8%)	<0.001
Disagree	6 (2.2%)	2 (1.6%)	
Neutral	83 (31.0%)	33 (26.4%)	
Agree	147 (54.9%)	28 (22.4%)	
Total agreement	28 (10.4%)	61 (48.8%)	
Item 15—To what extent do you agree that the information provided by AI is easy to understand?			
Total disagreement	4 (1.5%)	1 (0.8%)	<0.001
Disagree	3 (1.1%)	2 (1.6%)	
Neutral	54 (20.1%)	20 (16.0%)	
Agree	172 (64.2%)	38 (30.4%)	
Total agreement	35 (13.1%)	64 (51.2%)	
Item 16—To what extent do you agree that AI helped you determine whether your dental problem was urgent?			
Total disagreement	12 (4.5%)	3 (2.4%)	<0.001
Disagree	16 (6.0%)	10 (8.0%)	
Neutral	106 (39.6%)	29 (23.2%)	
Agree	106 (39.6%)	23 (18.4%)	
Total agreement	28 (10.3%)	60 (48.0%)	
Item 17—To what extent do you agree that the use of AI may induce unnecessary anxiety regarding dental problems?			
Total disagreement	12 (4.5%)	5 (4.0%)	<0.001
Disagree	24 (9.0%)	13 (10.4%)	
Neutral	172 (64.2%)	48 (38.4%)	
Agree	49 (18.3%)	25 (20.0%)	
Total agreement	11 (4.1%)	34 (27.2%)	
Item 18—How did using AI influence the timing of your dental consultation?			
Scheduled sooner	40 (14.9%)	11 (8.8%)	0.220
Delayed	10 (3.7%)	4 (3.2%)	
Unchanged	218 (81.3%)	110 (88.0%)	
Item 19—To what extent do you agree that AI helped you formulate better questions for your dentist?			
Total disagreement	15 (5.6%)	7 (5.6%)	0.983
Disagree	7 (2.6%)	3 (2.4%)	
Neutral	64 (23.9%)	26 (20.8%)	
Agree	117 (43.7%)	57 (45.6%)	
Total agreement	65 (24.3%)	32 (25.6%)	
Item 20—Did you discuss the information obtained from AI with your dentist?			
Absent	138 (51.5%)	74 (59.2%)	0.160
Present	130 (48.5%)	51 (40.8%)	
Item 21—Overall, how would you rate the use of AI for dental problems?			
Confusing	113 (42.2%)	67 (53.6%)	0.204
Low usefulness	70 (26.1%)	23 (18.4%)	
Neutral	53 (19.8%)	21 (16.8%)	
Useful	23 (8.6%)	12 (9.6%)	
Very useful	9 (3.4%)	2 (1.6%)	

* Fisher’s Exact Test.

**Table 4:** Distribution of the participants according to the answers in the survey and living environment.

Response (No., %)	Living Environment		*p* *
	Rural	Urban	
Item 6—How often have you used AI to obtain information related to dental health?			
Once	23 (50.0%)	94 (27.1%)	<0.001
A few times	18 (39.1%)	112 (32.3%)	
Frequently	3 (6.5%)	100 (28.8%)	
Very frequently	2 (4.3%)	41 (11.8%)	
Item 7—When did you use AI?			
Just before visit	28 (60.9%)	124 (35.7%)	<0.001
Only after visit	9 (19.6%)	24 (6.9%)	
Before and after visit	9 (19.6%)	199 (57.3%)	
Item 8—For which dental condition did you use AI?			
Dental pain	14 (30.4%)	131 (37.7%)	0.293
Cavity/sensitivity	10 (21.7%)	75 (21.6%)	
Gums/periodontal issues	9 (19.6%)	79 (22.8%)	
Oral lesions	1 (2.2%)	15 (4.3%)	
Other	12 (26.1%)	47 (13.5%)	
Item 9—What was the purpose of using AI?			
Diagnostic	12 (26.1%)	174 (50.1%)	0.005
Severity assessment	8 (17.4%)	59 (17.0%)	
Emergency consultation	1 (2.2%)	5 (1.4%)	
At home temporary solutions	19 (41.3%)	67 (19.3%)	
Treatment options	6 (13.0%)	42 (12.1%)	
Item 10—What type of information did you send to the AI?			
Only symptoms details	40 (87%)	258 (74.4%)	0.124
Intraoral photos	2 (4.3%)	47 (13.5%)	
Dental X-rays	2 (4.3%)	6 (1.7%)	
Personal ID	0 (0.0%)	2 (0.6%)	
All of the above	2 (4.3%)	34 (9.8%)	
Item 11—Have you used AI for the interpretation of dental images?			
On X-rays	0 (0.0%)	47 (13.5%)	<0.001
On intraoral photos	4 (8.7%)	6 (1.7%)	
Both	2 (4.3%)	55 (15.9%)	
Did not used images	40 (87.0%)	239 (68.9%)	
Item 12—What was AI mainly used for?			
General information	27 (58.7%)	244 (70.3%)	0.047
Preliminary orientation	7 (15.2%)	47 (13.5%)	
Suspicion confirmation	8 (17.4%)	50 (14.4%)	
Substitution of medical opinion	4 (8.7%)	6 (1.7%)	
Item 13—How would you evaluate the information provided by AI?			
Completely wrong	0 (0.0%)	2 (0.6%)	0.611
Partially wrong	2 (4.3%)	7 (2.0%)	
Unclear	7 (15.2%)	47 (13.5%)	
Mostly correct	26 (56.5%)	185 (53.3%)	
Very clear and useful	11 (23.9%)	106 (30.5%)	
Item 14—To what extent do you agree that AI provides useful information about dental conditions?			
Total disagreement	0 (0.0%)	5 (1.4%)	0.133
Disagree	3 (6.5%)	5 (1.4%)	
Neutral	17 (37.0%)	99 (28.5%)	
Agree	18 (39.1%)	157 (45.2%)	
Total agreement	8 (17.4%)	81 (23.3%)	
Item 15—To what extent do you agree that the information provided by AI is easy to understand?			
Total disagreement	1 (2.2%)	4 (1.2%)	0.076
Disagree	0 (0.0%)	5 (1.4%)	
Neutral	15 (32.6%)	59 (17.0%)	
Agree	23 (50.0%)	187 (53.9%)	
Total agreement	7 (15.2%)	92 (26.5%)	
Item 16—To what extent do you agree that AI helped you determine whether your dental problem was urgent?			
Total disagreement	3 (6.5%)	12 (3.5%)	0.345
Disagree	4 (8.7%)	22 (6.3%)	
Neutral	16 (34.8%)	118 (34.0%)	
Agree	17 (37%)	112 (32.3%)	
Total agreement	6 (13.0%)	83 (23.9%)	
Item 17—To what extent do you agree that the use of AI may induce unnecessary anxiety regarding dental problems?			
Total disagreement	5 (10.9%)	12 (3.5%)	0.135
Disagree	5 (10.9%)	32 (9.2%)	
Neutral	20 (43.5%)	200 (57.6%)	
Agree	10 (21.7%)	64 (18.4%)	
Total agreement	6 (13.0%)	39 (11.2%)	
Item 18—How did using AI influence the timing of your dental consultation?			
Scheduled sooner	9 (19.6%)	42 (12.1%)	0.001
Delayed	6 (13.0%)	8 (2.3%)	
Unchanged	31 (67.4%)	297 (85.6%)	
Item 19—To what extent do you agree that AI helped you formulate better questions for your dentist?			
Total disagreement	5 (10.9%)	17 (4.9%)	0.003
Disagree	2 (4.3%)	8 (2.3%)	
Neutral	15 (32.6%)	75 (21.6%)	
Agree	21 (45.7%)	154 (44.4%)	
Total agreement	3 (6.5%)	93 (26.8%)	
Item 20—Did you discuss the information obtained from AI with your dentist?			
Absent	41 (89.1%)	140 (40.3%)	<0.001
Present	5 (10.9%)	207 (59.7%)	
Item 21—Overall, how would you rate the use of AI for dental problems?			
Confusing	3 (6.5%)	8 (2.3%)	<0.001
Low usefulness	6 (13.0%)	29 (8.4%)	
Neutral	13 (28.3%)	62 (17.9%)	
Useful	18 (39.1%)	75 (21.6%)	
Very useful	6 (13.0%)	173 (49.9%)	

* Fisher’s Exact Test.

**Table 5:** Summary of the most clinically relevant associations identified in the study.

Key Finding	Stratifying Variable	Contrast (Subgroups)	*p* *
Sharing of visual data (intraoral photos) with AI	Age	45–54: 44.4% (*n* = 40) vs. 18–24: 4.5% (*n* = 3)	<0.001
Sharing of visual data (intraoral photos/combined data) with AI	Gender	Female: 17.2% (*n* = 46) vs. Male: 2.4% (*n* = 3)	<0.001
Use of AI as a substitute for professional opinion	Living environment	Rural: 8.7% (*n* = 4) vs. Urban: 1.7% (*n* = 6)	0.047
Perception that AI may induce unnecessary anxiety (high/very high agreement)	Gender	Male: 27.2% (*n* = 34) vs. Female: 4.1% (*n* = 11)	<0.001
Delay of dental consultation after AI use	Living environment	Rural: 13.0% (*n* = 6) vs. Urban: 2.3% (*n* = 8)	0.001
Discussion of AI-derived information with the dentist	Living environment	Urban: 59.7% (*n* = 207) vs. Rural: 10.9% (*n* = 5)	<0.001
Frequent AI use	Age	18–24: 56.1% (*n* = 37) vs. ≥55: 4.5% (*n* = 1)	<0.001

* Fisher’s exact test.

## Data Availability

The data presented in this study are available on request from the corresponding author. The data are not publicly available due to privacy reasons.
